# Synthetic lethality in cancer: a protocol for scoping review of gene interactions from synthetic lethal screens and functional studies

**DOI:** 10.1186/s13643-025-02814-2

**Published:** 2025-04-08

**Authors:** Raashi Chauhan, Rama Rao Damerla, Vijay Shree Dhyani

**Affiliations:** 1 Department of Medical Genetics, Kasturba Medical College, Manipal, Manipal Academy of Higher Education, Manipal, Karnataka India; 2 Kasturba Medical College, Manipal, Manipal Academy of Higher Education, Manipal, Karnataka India

**Keywords:** Synthetic lethal, DNA damage repair pathways, CRISPR/Cas9, Cancer, Targeted therapy

## Abstract

**Background:**

Two genes are synthetically lethal if loss of function of either one of the two genes does not result in cell death, whereas loss of function of both genes together results in being detrimental to cell survival. This concept has been the basis for developing personalized, precision treatments, which can selectively damage tumor cells and minimize toxicity to normal tissues. Tumor cells often harbor mutations in genes involved in DNA repair pathways, forcing them to switch to alternative repair pathways, leading to chemotherapeutic resistance. These interactions, if targeted, could be synthetically lethal. We aimed to summarize synthetically lethal gene pairs that could be utilized to selectively target cancer cells and minimize side effects on normal tissues. The objective of this review is to study druggable synthetically lethal gene pairs for targeted cancer therapy that have been identified through various genetic screens and functional studies.

**Methods:**

A systematic literature search will be conducted to extract synthetically lethal gene pairs that can be specifically targeted to cancer cells. Owing to the relatively recent research pertaining to this field, the literature search will incorporate data from 1956. The search will be conducted on PubMed, Web of Science, Embase, and Scopus. The narrative approach will guide the analysis and synthesis of the results.

**Discussion:**

This review highlights scientific articles that report druggable synthetically lethal gene pairs by testing the efficacy of targeted inhibitors in clonogenic assays. These include research studies that identify synthetically lethal gene pairs detected through CRISPR screens by knocking out one or two genes within the same cell and testing the potency of inhibitors to specifically kill malignant cells.

**Systematic review registration:**

10.17605/OSF.IO/5BCW6.

## Background

Cancer is a complex heterogeneous disease characterized by uncontrolled cellular proliferation and invasion of neighboring tissues. One of the hallmarks leading to this uncontrollable growth is genomic instability caused by perturbation of the DNA maintenance machinery [1]. Numerous defects involving genetic changes in genes that produce proteins involved in DNA damage recognition, repair, and protection of DNA from mutagens have been reported [1]. These proteins and their functions in conferring genomic stability are good chemotherapeutic targets and radiation sensitizers [2]. There is now a greater emphasis on developing personalized, precision therapies that can selectively target tumor cells while minimizing toxicity to normal tissues. This can be achieved through selectively targeting tumor-specific genetic interactions, a concept known as synthetic lethality. A pair of genes is synthetically lethal if loss of function of either gene does not lead to cell death, whereas loss of both genes results in cell death [3]. Tumors harbor mutations in one or more DNA repair pathways, leading to a reliance on alternative pathways [4] Recent drug discovery efforts have focused on developing therapeutic approaches targeting these alternative pathways, leading to the selective killing of cancer cells by exploiting the concept of synthetic lethality. Numerous drug candidates, which have been developed on the basis of synthetic lethality, are now in clinical trials, whereas advances in biotechnology and genomics are leading to the identification of novel synthetic lethal interactions [5].

DNA damage recognition proteins identify DNA damage, which can be caused by several exogenous and endogenous factors [6, 7]. This process also determines the choice of pathway for DNA repair. Therefore, to maintain genomic stability, mammalian cells have evolved highly conserved DNA repair mechanisms to correct DNA damage [3, 8]. There are six key DNA repair pathways that are involved depending on the type of DNA lesion; however, a significant crossover exists between the effector proteins in each pathway [2]. These pathways include base excision repair (BER) for single-stranded breaks [9], nucleotide excision repair for bulky lesions and cross links [10, 11], and mismatch repair (MMR) for single nucleotide mutations such as substitutions, insertions, and deletions [12, 13]. DNA double-stranded breaks (DSBs) are repaired through nonhomologous end joining (NHEJ) and resection-mediated repair through homologous recombination (HR), alternative NHEJ (Alt-NHEJ) [14], and single-stranded annealing (SSA) [15]. The choice of pathway is based on multiple parameters, one of the most important factors being the phase of the cell cycle. Defective DNA repair leads to genomic instability, which could promote neoplastic transformation and subsequent carcinogenesis [16].

Many studies now focus on identifying novel synthetic lethal targets through CRISPR screens. Xu Feng et al. performed a genome-wide CRISPR screen for knockout of loss-of-function tumor suppressor genes in isogenic cell lines and revealed several synthetic lethal interactions. They discovered that a tumor suppressor gene has the potential to serve as an anticancer target for cancers that are deficient in another tumor suppressor gene. This study revealed a synthetic lethal relationship between *TSC2* and *STK11*, between* KDM5C* and *BAP1*, and between *SMARCA4* or *ARID1A* and *PTEN* [17]. Another study by Zhou Z et al. conducted a short hairpin RNA (shRNA) library screen followed by next-generation deep sequencing and discovered that *PRKDC* was synthetically lethal in human lung fibroblasts overexpressing *MYC* [18]. These gene interactions can be used as drug targets and exploited for targeted cancer therapy.

One of the most common examples of drugs that target synthetic lethal gene interactions is poly(ADP‒ribose) polymerase (PARP) inhibitors (PARPis), which have been approved for treating breast, ovarian, pancreatic, and prostate cancers with loss-of-function BRCA1/2 mutations [19]. Both BRCA1 and BRCA2 are responsible for homologous recombination, an error-free DNA repair pathway used for repairing DSBs. HR deficiency in cancer cells caused by deleterious mutations in BRCA1/2 can be selectively targeted by inhibiting PARP, a class of enzymes involved in repairing different types of DNA damage. Inhibition or trapping of PARP on DNA leads to DSB formation, which further requires HR proteins such as BRCA1/2 for repair. Therefore, in HR-deficient cells, PARPis can lead to the accumulation of DSBs, leading to genomic instability and cell death [20]. This synthetic lethal interaction between PARPis and BRCA1/2 deficiency has been exploited to treat several cancers with HR deficiency.

In the proposed scoping review, we aim to summarize and explore druggable synthetically lethal pairs that could be potential anticancer drug targets. A preliminary search of PubMed, Google Scholar, Web of Science, Embase, Scopus, and JBI Evidence Synthesis was conducted in January 2025, and no current or underway systematic reviews or scoping reviews on the topic were identified. This study reports research articles that have identified synthetically lethal gene pairs via clonogenic assays such as shRNA knockdown and CRISPR knockout screens for one or two genes within the same cell and tests the potency of the inhibitors to specifically target cancerous cells.

## Methods and design

### Aim

To study druggable synthetically lethal gene pairs in targeted cancer therapy, which have been identified through various genetic screens and functional studies.

### Participants

NA

### Concept

This scoping review will examine the concept of synthetic lethality. Tumors often harbor mutations in one or more DNA repair pathways, leading to a reliance on alternative pathways [4]. Therefore, inhibition of an important alternative pathway can lead to a nonviable accumulation of unrepaired DNA and subsequent apoptosis. Defects in DNA maintenance can increase the susceptibility of cancer cells to chemotherapy and radiotherapy, but over time, these cells may develop resistance. To counter this, DNA damage repair therapies use chemosensitizers and exploit synthetic lethality to selectively target cancer cells while minimizing side effects. Compared with conventional therapy, this approach is promising because of its efficiency and reduced side effects, making it a highly recognized strategy in cancer treatment. Identification and targeting of synthetic lethal interactions could help develop precise and personalized cancer therapies while minimizing adverse effects, as observed in cases of traditional chemotherapy or radiation therapy.

### Context

This review focuses on how genes involved in DNA repair pathways compensate for each other and lead to chemotherapy resistance. We also explore the concept of synthetic lethality by summarizing the combinations of genetic pathways and drug interactions that have been identified through genetic or mutational screens and other functional studies and correlate their importance with the potential of being utilized as novel and future drug targets for cancer treatment.

### Types of sources

This scoping review considers experimental study designs, which include genetic screens and functional studies. This review will also examine observational study designs and quantitative studies.

### Search strategy

Published studies in English until January 2025 will be searched by combining free text terms and database-specific controlled vocabulary related to synthetic lethality and cancer using Boolean operators (OR, AND) and appropriate field tags (All felids, Title, Abstract). The search will be carried out on Medline (PubMed), Embase, Web of Science, and Scopus. Additionally, Google Scholar and the reference list of all included sources of evidence will also be screened for locating additional studies. The text words present in the title, abstracts, and index terms of the relevant articles were used to develop the search strategy. The initial search strategy was developed for Medline (PubMed) and customized according to the respective databases till January 2025 (Table 2 in Appendix).

### Study/source of evidence selection

After the search, all the selected citations will be aggregated and uploaded to Rayyan, and duplicates will be removed [21]. The articles will be subsequently screened by two independent reviewers on the basis of the inclusion criteria of the review. Articles that do not meet the inclusion criteria will be excluded from the study. Disagreements between the two reviewers will be resolved by discussion with a third reviewer. The results of the search and inclusion process will be reported completely in the final scoping review and presented in the Preferred Reporting Items for Systematic Reviews and Meta-analyses extension for scoping review (PRISMA-ScR) flow diagram [22]*.*

### Data extraction

Data will be extracted from papers included in the scoping review by two independent reviewers with the data extraction tool developed and pilot tested by the reviewers. Disagreements will be discussed with a third reviewer to resolve any differences. Attempts to contact the authors of the included studies will be made if there are any missing or additional data needed. The data extracted will include specific details about the concept, context, study methods, and key findings relevant to the review questions. The draft of the data extraction is attached in Table 3 in Appendix.

### Data analysis and presentation

The data will be presented in tabular and diagrammatical formats. The analysis will be performed on the basis of information pertaining to synthetic lethal gene pairs that have been discovered through CRISPR screens and other functional studies. This analysis will also include chemotherapeutic drugs that target synthetically lethal gene pairs.

It will encompass drugs that are used for treating patients and those that are undergoing clinical trials. For example, olaparib is used as a chemotherapeutic drug to treat breast and ovarian cancer patients. It is a PARP inhibitor and causes the apoptosis of cancer cells, which results from a *BRCA1/2* mutation. *PARP* and *BRCA1/2* are synthetically lethal gene pairs, which implies that loss of function of both of these genes can lead to cell death [23].

The diagrammatical format represents the strategy used to congregate all the information. We will also utilize narrative summaries to report additional details, such as study citations and methodological details.

## Discussion

This review aims to summarize synthetically lethal gene pairs that may be possible therapeutic targets for precision medicine in cancer. Tumor cells harbor mutations in genes that are responsible for DNA repair and force them to rely on alternate pathways, which leads to chemotherapeutic resistance [2, 4]. Normal cells possess intact pathways to repair such damage, leading to the selective killing of cancer cells. The definition of synthetic lethality has been expanded to encompass pharmacological inhibition of one gene product with inactivation of the other in cancer cells [24].

The perturbation of one of the synthetically lethal gene pairs by chemical or genetic inhibition in cancer cells with an already defective gene partner can be detrimental to survival [3]. Large-scale CRISPR-based screens have been utilized to identify numerous synthetic lethal gene pairs, many of which are in drug development pipelines. A few examples of candidate genes are *BRCA1, BRCA2, TP53, KRAS, MYC, ATM, ATR,* and *CHK1*, which are targeted for specific cancer types and are currently at different stages in clinical trials [25–30]. We also examined synthetic lethal gene interaction data from previously published genetic screens, such as *ARID1A, TSC2, KDM5C, SMARCA4, PTEN, WEE1, BRG1, WRN, TP53, CCNE1, BRCA1/2, SF3B1, and KRAS,* which are presumed to play a role in multiple cancers [17, 31–36], as detailed in Table 1.

**Table 1:** Synthetic lethal gene pairs and associated cancers identified from genetic screens and functional studies

S. No.	Gene	Synthetic lethal partner	Cancer type
1	ARID1A	TEAD1	Hepatocellular carcinoma [32]
2	TSC2	STK11	Non-small cell lung cancer [17]
3	KDM5C	BAP1	Diverse cancers [17]
4	SMARCA4	ARID1A	Diverse cancers such as ovarian cancer, breast cancer, lung adenocarcinoma, clear cell renal cell carcinoma [17]
5	SMARCA4	PTEN	Cowden syndrome, hamartomas, and other diverse cancers [17]
6	ARID1A	ARID1B	Neuroblastoma and other diverse cancers [17]
7	ARID1A	EZH2	Breast cancer, prostate cancer, and other diverse cancers [17]
8	PTEN	CHD1	Cowden syndrome, hamartomas and other diverse cancers [17]
9	WEE1	ATR	Breast cancer [28, 30]
10	BRG1	PTEN	Prostate cancer [33]
11	WRN	MMR genes	Colon cancer [34]
12	TP53	MDM2	TP53 is a critical tumor suppressor gene that is mutated in more than half of the human cancers [25]
13	TP53	MYC	TP53 is a critical tumor suppressor gene that is mutated in more than half of the human cancers [31]
14	CCNE1	PKMYT1	High-grade serous ovarian cancer, uterine tumors, and gastro-oesophageal cancers [35]
15	BRCA1/2	CIP2A	BRCA1/2 mutated cancers such as breast and ovarian cancer [26]
16	BRCA2	APEX2 and FEN1	BRCA2 mutated cancers such as breast and ovarian cancer [36]
17	SF3B1	BRCA1/2	BRCA2 mutated cancers such as breast and ovarian cancer [28]
18	KRAS	NOP56	Lung cancer [29]

Periodic exposure of tumors to radiation and chemotherapeutic drugs could confer resistance to therapy, leading to poorer clinical outcomes. To overcome this setback, recent drug discovery initiatives have resorted to the development of developing radiosensitizers and chemosensitizers through the concept of synthetic lethality. This strategy for precision medicine aims to selectively target cancer cells while minimizing adverse effects. The application of synthetic lethality for DNA damage repair inhibitors has gained immense recognition because of the complexity of multiple pathways compensating for each other, contributing to drug resistance. This seems to be a promising approach because it exploits this concept against cancer cells and makes it more selective, efficient, and lethal for malignant cells with fewer side effects than conventional therapy.

This review focuses on summarizing synthetically lethal gene pairs and explores whether their interactions can be utilized for targeted cancer therapy. This study provides a resource summarizing several synthetically lethal gene pairs for further functional studies with respect to expanding these drugs into clinical trials and testing their efficacy.

## Data Availability

Not applicable.

## Appendix

**Table 2:** Search strategy

Database	Date of search	Search strategy
Pubmed	14–01-2025	(((((((((((synthetic) AND (lethality)) OR (synthetic lethal mutations)) AND (neoplasms)) AND (DNA repair)) OR (DNA repair deficiency disorders))) OR (crisprassociated protein 9)) OR (crisprcas systems)) OR (crispri screens)) OR (Antitumor Drug Screening Assays)) OR (RNA interference)
Scopus	14–01-2025	(TITLE-ABS-KEY (synthetic AND lethality OR synthetic AND lethal AND mutations) AND TITLE-ABS-KEY (neoplasms) AND TITLE-ABS-KEY (dna AND repair OR dna AND repair AND deficiency AND disorders) OR TITLE-ABS-KEY (crispr AND associated AND protein 9 OR crispr AND cas AND systems OR crispr AND screens) OR TITLE-ABS-KEY (drug AND screening AND assays, AND antitumor) OR TITLE-ABS-KEY (rnai) AND TITLE-ABS-KEY (cancer))
Embase	14–01-2025	((“synthetic lethality”/exp OR “synthetic lethality” OR “synthetic lethal mutation”/exp OR “synthetic lethal mutation”) AND (“neoplasm”/exp OR “neoplasm”) AND (“dna repair”/exp OR “dna repair” OR “disorders of dna synthesis and repair”/exp OR “disorders of dna synthesis and repair”) OR “crispr associated protein”/exp OR “crispr associated protein” OR “crispr cas system”/exp OR “crispr cas system” OR “crispr screens” OR ((“crispr”/exp OR crispr) AND screens) OR “drug screening”/exp OR “drug screening”) AND (“rnai”/exp OR “rnai”) AND (“malignant neoplasm”/exp OR “malignant neoplasm”)
Web of Science	14–01-2025	(((((((((((synthetic) AND (lethality)) OR (synthetic lethal mutations)) AND (neoplasms)) AND (DNA repair)) OR (DNA repair deficiency disorders))) OR (crisprassociated protein 9)) OR (crisprcas systems)) OR (crispri screens)) OR (Antitumor Drug Screening Assays)) OR (RNA interference)

**Table 3:** Data extraction instrument

**General information about the study**	
Authors	Zizhi Tang Jun Chen, Ming Zeng, Xiaojun Wang, Chang Guo, Peng Yue, Xiaohu Zhang, Huiqiang Lou, Dezhi Mu, Daochun Kong, Antony M. Carr, and Cong Liu
Year of publication	23rd May 2022
Country in which it was conducted	China and UK
**Details of the study**	
Aim	To characterize the potential role of ENDOD1 in DNA repair
Context	This study uncovers the role of ENDOD1 in the context of DNA repair pathway and its synthetically lethal interaction with TP53 which is a well-known tumor suppressor gene. It also explores the possibility of ENDOD1 serving as a specific target against cancer cells in view of its synthetic lethal relationship with TP53
Type of study	In vitro study
Method	Cell proliferation and viability; Clonogenic assays: Comet Assay to assess DNA repair efficiency; CCK8 colorimetry to show the siRNA knock out efficiency; Measurement for PARP-DNA complex; Immunofluorescence Immunofluorescentstaining and Western Blotting; Flow cytometry and fluorescent-activated cell sorting (FACS); Animal, biopsies and histochemical staining
Key findings	Identified ENDOD1 as a potential wide-spectrum and cancer-specific target for SL drug discovery

## Abbreviations

PRISMA-ScR: Preferred Reporting Items for Systematic Review and Meta-Analysis Extension for Scoping Reviews
PRISMA: Preferred Reporting Items for Systematic Reviews and Meta-Analyses
CRISPR: Clustered Regularly Interspersed short palindromic repeats
PARP: Poly (ADP‒ribose) polymerase
PARPi: PARP inhibition
PARPis: PARP inhibitors
MMR: Mismatch repair:
HR: Homologous recombination
NHEJ: Nonhomologous end joining
Alt-NHEJ: Alternative NHEJ
SSA: Single-stranded annealing
DSB: Double-stranded breaks
